# In this month’s Bulletin

**DOI:** 10.2471/BLT.25.001025

**Published:** 2025-10-01

In the editorial section, Nicola Abrescia et al. (578) detail associations between climate change and the incidence of respiratory infections in humans. Shin Ling Wu (579) explores policy lessons from a vaping ban in Singapore.

## Algeria, Bangladesh, Central African Republic, Chad, Democratic Republic of the Congo, Dominican Republic, Fiji, Gambia, Ghana, Guinea-Bissau, Guyana, Honduras, Iraq, Kiribati, Lao People’s Democratic Republic, Lesotho, Madagascar, Malawi, Mongolia, Nepal, occupied Palestinian territory including east Jerusalem, Samoa, Sao Tome and Principe, Sierra Leone, Suriname, Togo, Tunisia, Tuvalu, Zimbabwe

### Water quality and child nutrition

Dung Duc Lea and Long Thanh Giang (582–591) review the evidence from 29 countries.

## Democratic Republic of the Congo

### Anticipating cholera

Pei Shan Loo et al. (607–618) identify areas at greater risk of outbreaks.

## Region of Africa

### Treatments for Parkinson disease

Natasha Fothergill-Misbah et al. (626–634) examine national essential medicines lists. 

## Global

### Digitialization of the health sector

Lena Kan et al. (592–606) study primary health care performance in 109 countries and territories.

### Ethical clinical trial design

Roger J Lewis et al. (619–625) propose methods to account for differences in treatment effects. 

### HIV prevention, treatment and care

Erin K Ferenchick et al. (635–637) argue for the integration of mental health services.

### WHO Pandemic Agreement 

WooJung Jon (638–640) considers implementation requirements.

